# What should be the best dialysis catheter lock in critically ill patients?

**DOI:** 10.1186/s13054-019-2640-1

**Published:** 2019-10-30

**Authors:** Patrick M. Honore, Leonel Barreto Gutierrez, Sebastien Redant, Keitiane Kaefer, Andrea Gallerani, David De Bels

**Affiliations:** ICU Department, Centre Hospitalier Universitaire Brugmann-Brugmann University Hpospital, Place Arthur Van Gehuchtenplein, 4, 1020 Brussels, Belgium

In the recent years, several studies including a meta-analysis have assessed the question concerning the best dialysis catheter lock. We sought to summarize these trials in order to give some guidelines to the clinicians. In a recent meta-analysis, Zhong et al. did not demonstrate any superiority of heparin locked over normal saline for maintaining the patency of central venous catheter including one’s dialysis [1]. To avoid catheter occlusion, thrombosis, and catheter-related bloodstream infection (CRBSI), proper flushing and locking are considered to be the primary interventions [2]. A high antimicrobial concentration lock should be used to overcome the relative resistance of bacteria in the catheter biofilm. Antibiotic locks decrease the risk of long-term hemodialysis infection, but, when used repeatedly, may promote the selection of resistant organisms [3]. Therefore, clinicians were looking to other alternatives such as citrate. Indeed, citrate concentrated at 1 to 4% exerts only an anticoagulant effect by its ability to chelate calcium [3]. Highly concentrated citrate up to 46.7% exerts additional effects like the inhibition of catheter colonization [3]. Randomized controlled trials (RCTs) comparing heparin versus citrate lock solutions at 46.7% have been relatively limited [3]. Indeed, FDA has banned citrate solutions with a concentration higher than 4% because of the accidental risk of major drop in ionized calcium and subsequent cardiac arrest [3]. Nevertheless, Parienti et al. performed a prospective quasi-experimental study comparing citrate (CL) at 46.7% vs heparin locks or saline [3]. CL was associated with less catheter colonization possibly by impeding biofilm. The use of CL was also associated with less catheter dysfunction. The higher rate of catheter dysfunction found in the saline group as opposed to the CL group was in accordance to Hermite et al. [4]. CL at 46.7% was not associated with higher mortality [3]. However, the reduction of catheter dysfunction was not associated with a longer catheter duration [3]. Comparing citrate 4% to heparin locks by Quenot et al. [5] found no differences in the duration of event-free survival of the first non-tunneled hemodialysis catheter. Catheter thrombosis, catheter-related infections (CRI), and adverse events were not statistically different between the two groups. In conclusion, it seems that CL 46.7% should be the best option for dialysis catheter locks in the intensive care unit (ICU). CL has less catheter colonization, less catheter dysfunction, but no superior catheter duration [5]. Future RCTs are obviously needed to confirm these findings.

## Data Availability

Not applicable.

## Abbreviations

CRBSI: Catheter-related bloodstream infection
NS: Normal saline
CVC: Central venous catheter
CRI: Catheter-related infection
RCT: Randomized controlled study
CL: Citrate lock
ICU: Intensive care unit

## References

[1] Zhong L, Wang HL, Xu B, Yuan Y, Wang X, Zhang YY (2017). Normal saline versus heparin for patency of central venous catheters in adult patients - a systematic review and meta-analysis. Crit Care.

[2] Goossens GA (2015). Flushing and locking of venous catheters: available evidence and evidence deficit. Nurs Res Pract.

[3] Parienti JJ, Deryckère S, Mégarbane B, Valette X, Seguin A, Sauneuf B (2014). Quasi-experimental study of sodium citrate locks and the risk of acute hemodialysis catheter infection among critically ill patients. Antimicrob Agents Chemother.

[4] Hermite L, Quenot J-P, Nadji A, Barbar SD, Charles P-E, Hamet M (2012). Sodium citrate versus saline catheter locks for non-tunneled hemodialysis central venous catheters in critically ill adults: a randomized controlled trial. Intensive Care Med.

[5] Quenot JP, Helms J, Bourredjem A, Dargent A, Meziani F, Badie J (2019). Trisodium citrate 4% versus heparin as a catheter lock for non-tunneled hemodialysis catheters in critically ill patients: a multicenter, randomized clinical trial. Ann Intensive Care.

